# Research Integrity Attitudes and Behaviors are Difficult to alter: Results from a ten Year Follow-up Study in Norway

**DOI:** 10.1177/15562646221150032

**Published:** 2023-01-05

**Authors:** Bjørn Hofmann, Magne Thoresen, Søren Holm

**Affiliations:** 1 Centre of Medical Ethics, University of Oslo, Oslo, Norway; 2 87362 Department of Health Sciences, Norwegian University of Science and Technology, Gjøvik, Norway; 3 Institute of Basic Medical Sciences, 60504 Faculty of Medicine, University of Oslo, Oslo, Norway; 4 Centre for Social Ethics and Policy, School of Law, 5292 University of Manchester, Manchester, UK

**Keywords:** knowledge, attitudes, practice, Norway, PhD, medicine, research integrity

## Abstract

*Background:* Research integrity has obtained much attention in research communities, but also in the general public. To improve research integrity is difficult as it involves complex systems of knowledge, attitudes, and practices. The objective of this study is to investigate the knowledge, attitudes, and practices of cohorts of PhD candidates at one faculty (of medicine) over time and compare this to finished PhDs of the same cohorts. *Material and method:* Researchers (n  =  186) awarded the degree PhD at the Faculty of Medicine at the University of Oslo in 2019 were invited to answer a questionnaire about knowledge, attitudes and actions related to scientific dishonesty. 94 responded (50.5%). The results were compared with results among first-year PhD candidates who responded to the same questionnaire during 2010–20 (n  =  536) and to those who finished PhDs in 2016 (n  =  86). *Results:* For the years 2010–2020 1.1% of the PhD candidates report to have engaged in severe scientific misconduct (FFP) while 0.9% report to have presented results in a misleading way. 2.3% report that they know of persons at their department who have engaged in FFP the last 12 months. In total 1.5% report to have experienced pressure to engage in severe scientific misconduct (FFP) while 2.1% report to have experienced pressure to present results in a misleading way. On average 12.8% report to have been exposed to unethical pressure concerning inclusion or ordering of authors during the last 12 months, and 28.8% report to have knowledge about their department's written policies about research integrity. While some attitudes improve over the years, attitudes in general are not much changed from 2010–2020. None of the PhDs that received a PhD from the Faculty of Medicine at the University of Oslo in 2019 reported to have engaged in FFT or having experienced pressure to do so.1.1% experienced pressure to present results in other misleading ways, while 26.6% of respondents had experienced unethical pressure in relation to authorship during the course of the PhD fellowship. 4.3% knew about someone at their department who had presented results in a misleading manner. Some attitudes were not in line with traditional conceptions of research integrity, but most agreed that their research environment displayed research integrity. *Conclusion:* This long-term follow up study shows that few PhD-candidates report to engage in severe scientific misconduct, that they experience little pressure to do so, and with some exceptions, attitudes in in line with good research integrity. However, pressure in relation to authorship is relatively common. There is some improvement in research integrity from PhD candidates to recently finished PhDs, but in general research integrity is stable over time.

## Introduction

A growing number of studies document breaches of scientific norms and a deficit in research integrity in a wide range of scientific fields. A recent systematic review and meta-analysis reports that 2.9% (95% CI 2.1–3.8%) of researchers have committed research misconduct in terms of either falsification, fabrication, or plagiarism (FFP) and that 12.5% (95%CI 10.5–14.7%) have committed at least one incidence of (unspecified) questionable research practices (QRP).7%). In addition, 15.5% (95% CI 12.4–19.2%) of researchers witnessed others who had committed at least one FFP and 39.7% (95% CI 35.6–44.0%) were aware of others who had applied at least one QRP (Xie, Wang, & Kong, 2021). Knowledge has also increased about factors that impact research integrity, such as pressure to publish, research funding, personality type, and the research environment (DuBois et al., 2013; Fanelli, 2010; Martinson, Crain, Anderson, & De Vries, 2009; Tijdink et al., 2016). Career level, disciplinary background and locations are documented to influence the prevalence of FFP and QRPs. For example, FFP and QRP are reported to be more prevalent in social sciences than in biomedical and interdisciplinary sciences (Xie et al., 2021).

Many measures have been taken to improve research integrity and to avoid FFP and QRP (Anderson et al., 2012; Forsberg et al., 2018). Despite increased attention and more accessible resources for the improvement of research integrity (Embassy of Good Science, https://embassy.science/wiki/Main_Page), there is little evidence of improvement (Fanelli, 2009; Xie et al., 2021). To improve, we need longitudinal data from the same institutions.

For example, few studies have followed the integrity of PhD candidates from the same institution or faculty over time or compared groups of researchers while they are PhD candidates and when they have finished their PhD. This study is therefore unique in two ways. First, it compares PhD candidates’ knowledge, attitudes, actions, and experiences with scientific dishonesty at the same faculty for one decade making it possible to detect development over time in one institution. Second, it compares the same aspects of research integrity when the PhD candidates have received their PhD making it possible to estimate the effect of studying for a PhD.

The corresponding research questions are: 1) How has the knowledge, attitudes, actions, and experiences with scientific dishonesty developed from 2010 to 2020 at the Faculty of Medicine at the University of Oslo? 2) How do these aspects of research integrity change after the candidates have finished their PhD?

While building on previous research (Hofmann, Myhr, & Holm, 2013; Hofmann & Holm, 2019; Holm & Hofmann, 2018), this article adds data to complete 10 years follow up and to compare PhD candidates with finished PhDs. This provides insights into how knowledge, attitudes, and practices change from research students to finished PhDs and whether they are connected to experiences in the research environment they have worked in. This is important information for forming a strategy for improving the integrity of researchers, especially if integrity habits are formed early in the socialization as a researcher.

## Material and Methods

All persons awarded the degree PhD at the Faculty of Medicine at the University of Oslo in 2019 with a traceable personal e-mail address were invited to participate in a two-page survey. This was done by using the university's official list of persons awarded a PhD, tracing these candidates by available address lists and recent publications. In addition, PhD-candidates attending obligatory introductory courses on research methodology in some of the years 2010–2019 (see Table 1 for specific years) were invited to participate in an anonymous paper survey, and PhD-candidates attending the courses in 2020 were invited to participate in an anonymous digital survey.

**Table 1.** Overview of Questionnaires distributed and returned along with answers to background to respondents to completed PhDs in 2019 and PhD students during the period 2010–2020 (2010, 2014, 2015, 2016, 2017, 2018, 2019, 2020).

Question / Background information	PhD from Oslo 2019	PhD students Oslo 2010–20
Returned/distributed (n)	94/186	536/752
Response rate (%)	50.54	71.28
Undergraduate studies in Norway, n (%)	65 (69.1)	328 (57.9)
Doing Clinical / Basic / Other research (%)	50/30.9/19.1	55.7/29.6/14.6
Lectures or courses in science ethics as part of undergraduate studies (Yes/No/I do not remember) (%)	77.7/12.8/9.5	67.6/23.2/9.2

The survey consisted of four parts. The first part, on knowledge and practices, was developed and first applied in Lund, Sweden (Nilstun, Lofmark, & Lundqvist, 2010). The second part, on attitudes was developed in the USA (Kalichman & Friedman, 1992) and validated (Holm & Hofmann, 2017). A third part was developed to investigate environmental factors. Various parts of the survey have previously been used in Norway (Hofmann, 2016; Hofmann, Helgesson, Juth, & Holm, 2015; Hofmann & Holm, 2016; Hofmann et al., 2013), Croatia (Holm & Hofmann, 2018; Ljubenković, Borovečki, Ćurković, Hofmann, & Holm, 2021), Sweden (Nilstun et al., 2010), and Denmark (Hofmann et al., 2020; Jensen, Kyvik, Leth-Larsen, & Eriksen, 2018).

Questions about facts or actions were scored as Yes/No/ Uncertain. A Likert-type scale was used for questions about attitudes (strongly disagree/disagree/neither disagree or agree/agree/strongly agree), coded from 1 (strongly disagree) to 5 (strongly agree) in the computation of the Kalichmann scores in Table 2. Table 1 shows the overview of the number of questionnaires distributed and returned together with answers about the background of the respondents.

**Table 2.** Average (SD) attitudes, Kalichmann-scores for PhDs in Oslo 2016, 2019 and first-year research fellows 2010–20.

Question	PhD from Oslo 2019 N = 94	PhD from Oslo 2016 N = 71	First-year research fellow 2010–2020 N = 526	Kalichman sub-scale scores^1^
It is never appropriate to report experimental data that have been created without actually having conducted the experiment.	4.56 (1.03)	4.65 (0.83)	4.60 (0.95)	General attitude*** PhD from Oslo 2019: 27.80 (3.61) PhD from Oslo 2016: 28.20 (2.51) First-year research fellow 2010–20: 26.86 (3.48) p = 0.001
It is never appropriate to alter experimental data to make an experiment look better than it actually was.	4.84 (0.55)	4.90 (0.38)*	4.76 (0.64)	
It is never appropriate to try a variety of different methods of analysis until one is found that yields a result that is statistically significant.	4.15 (0.96)	4.32 (0.92)***	3.80 (1.04)	
It is never appropriate to take credit for the words or writing of someone else.	4.85 (0.62)	4.78 (0.51)	4.66 (0.72)	
It is never appropriate to take credit for the data generated by someone else.	4.71 (0.71)	4.83 (0.51)***	4.52 (0.84)	
It is never appropriate to take credit for the ideas generated by someone else.	4.68 (0.71)	4.71 (0.59)**	4.48 (0.83)	
If you were confident of your findings, it is acceptable to selectively omit contradictory results to expedite publication.	1.65 (1.00)	1.89 (1.29)	1.97 (1.24)	Personal attitude (reverse scored) PhD from Oslo 2019: 9.14 (1.72) PhD from Oslo 2016: 8.56 (2.48) First-year research fellow 2010–20: 8.50 (2.28) P = 0.04
If you were confident of your findings, it is acceptable to falsify or fabricate data to expedite publication.	1.21 (0.84)	1.54 (1.34)	1.55 (1.25)	
It is more important that data reporting be completely truthful in a publication than in a grant application.	2.56 (1.28)	2.61 (1.38)	2.86 (1.33)	
If you witness someone committing research misconduct, you have an ethical obligation to act.	4.31 (0.64)	4.45 (0.71)*	4.28 (0.75)	Attitude whistle blowing* PhD from Oslo 2019: 12.36(1.92) PhD from Oslo 2016: 12.82 (2.02) First-year research fellow 2010–2020: 12.27 (2.10) P = 0.10
If you had witnessed a co-worker or peer committing research misconduct, you would be willing to report that misconduct to a responsible official.	4.10 (0.70)	4.24 (0.76)*	4.03 (0.78)	
If you had witnessed a supervisor or principal investigator committing research misconduct, you would be willing to report that misconduct to a responsible official.	3.96 (0.84)	4.14 (0.82)	3.96 (0.84)	
If fabricated data are discovered in a published paper, all co-authors must equally share in the blame.	3.31 (1.05)	3.51 (1.25)	3.42 (1.09)	Attitude punishment PhD from Oslo 2019: 6.06 (1.96) PhD from Oslo 2016: 6.36 (2.34) First-year research fellow 2010–20: 6.27 (2.02) P = 0.59
If fabricated data are discovered in a published paper, all co-authors must receive the same punishment.	2.76 (1.06)	2.86 (1.29)	2.86 (1.08)	

^1^ For the derivation of the sub-scales see (Kalichman, 2005).

For the respondents in 2020, 69.0% were female, 45.2% were doing clinical research, 40.5 were doing basic research, and 14.3% were doing other research, e.g., health services research or social science research. 66.7% had been doing research less than one year, while 33.3% had been doing research between one and two years. 73.8% had participated in previous courses on research ethics, and 59.9% had their Masters’ degree from Norway. While Table 1 presents the demographics for all years together, the demographics for the other years (2010–2019) can be found in the publications (Hofmann et al., 2015; Hofmann & Holm, 2019; Hofmann et al., 2013).

We compared the results of the PhDs who had finished 2019 with the results of the research students who responded to the same questionnaire in 2010–20 as first year research students in order to examine whether studying for the PhD had influenced attitudes. Data from PhD-candidates in 2020 were collected digitally (due to the pandemic), all other data collection rounds were paper based. We also compared these results with the results from the PhDs who had finished in 2016 who are described in detail in (Hofmann & Holm, 2019).

Statistical analyses are performed in IBM SPSS Statistics Version 27. We applied mainly descriptive analyses. Changes over time (2010 to 2020) are analyzed by a Monte Carlo exact test for nominal data and a Kruskall-Wallis test for ordinal data. Kalichman scores are analyzed by Analysis of Variance (ANOVA). We show results with a significance level of 5%. The study is reported to the Norwegian Data Protection Official for Research (NSD, Project No. 55147). Participation was voluntary and it is not possible to identify individuals from the results. Consent was obtained after informing (in writing and orally where possible) about the survey, about its anonymity, and it was made explicitly clear that participation was voluntary and that participants could withdraw (stop) at any point. Consent was given by answering the questions. No personal data traceable to individual participants was registered, and the study was thus not subject to Research Ethics Committee (or Institutional Review Board) approval, in accordance with Norwegian law.

## Results

### Trends from 2010 to 2020

In total 1.5% of the PhD candidates report to have experienced pressure to engage in severe scientific misconduct (Falsification, Fabrication or Plagiarism (FFP)) from 2010 to 2020. 2.1% report to have experienced pressure to present results in a misleading way.

1.1% report to have engaged in severe scientific misconduct (FFP) while 0.9% report to have presented results in a misleading way. 2.7% report that they know of persons at their department who have engaged in FFP the last 12 months.

In total 12.8% report to have been exposed to unethical pressure concerning inclusion or ordering of authors during the last 12 months, and less than one third (28.8%) report to have knowledge about their department's written policies about research integrity. If the number of respondents who are uncertain are added, most of these numbers double (and some triple).

**Figure 1.**
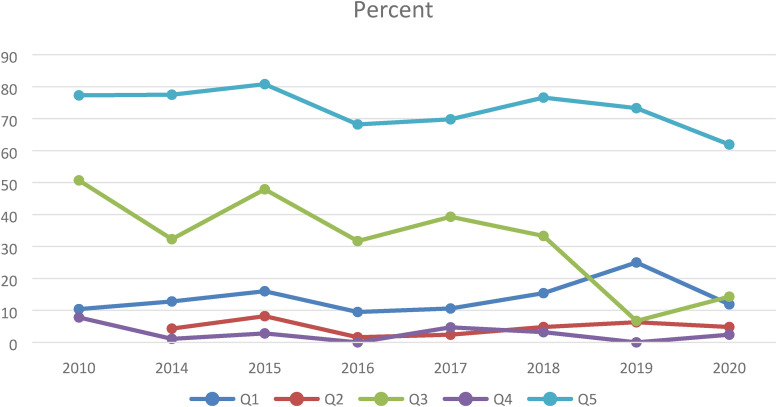
Development for answers to five specific questions. Q1 corresponds to those who report to have been exposed to unethical pressure concerning inclusion or ordering of authors during the last 12 months and Q2 are those who have answered ‘yes’ to the question “do you know about anyone at your department who during the last 12 months has presented results in some other misleading way?”. Q3 corresponds to those who do not strongly agree or agree that “it is never appropriate to try a variety of different methods of analysis until one is found that yields a result that is statistically significant.” Q4 are those who do not strongly agree or agree with the statement that “It is never appropriate to alter experimental data to make an experiment look better than it actually was.” Q5 are those who agree with the statement “If you had witnessed a supervisor or principal investigator committing research misconduct, you would be willing to report that misconduct to a responsible official.” The differences over time regarding Q3 are statistically significant (p < 0.001).

Overall, there is little change in the responses from 2010 to 2020. Figure 1 illustrates the development over time for specific issues.

Additionally, there were some changes in the PhD candidates’ knowledge about their departments’ written policies from 2010 to 2015 (which was the period where these questions were included in the survey). The candidates became more uncertain about whether the department had written policy about fabrication of data (43.4% in 2010 vs. 64.4% in 2015), handling of authorship (44.7% in 2010 vs. 65.3% in 2015), the use of funds (39.5% in 2010 vs. 67.1% in 2015), and about harassment (48.7% in 2010 vs. 65.3% in 2015), all differences being statistically significant.

### Integrity Amongst Finished PhDs Compared to PhD Candidates

187 persons are reported as having received a PhD from the Faculty of Medicine at the University of Oslo in 2019. We were able to identify 186 e-mail addresses of which 94 responded and completed the online questionnaire. 48% of the respondents still have research as a main activity. Demographic data for all responding PhDs and for the responding research students used for comparison can be found in Table 1.

None of the responding PhDs reported that they experienced pressure to fabricate, falsify or plagiarize data during doctoral work. The same was true for having experienced pressure to plagiarize entire publications, while one PhD (1.1%) experienced pressure to present results in other misleading ways.

However, 26.6% of respondents had experienced unethical pressure in relation to authorship during the course of the fellowship (In addition, 13.8% were uncertain). The PhDs also reported to have experienced pressure with respect to analysis (2.1%), and results (1.1%). In total, 6.4% had experienced unethical pressure in relation to other issues than authorship, if we include those who were uncertain.

On questions about their own practices as PhDs during their fellowships, 1.1% were unsure whether they had plagiarized publications and 1.1% were unsure whether they had presented results in a misleading way. No one reported having fabricated, falsified or plagiarized data.

1.1% of the PhDs reported to know about someone who had fabricated data at their department during their fellowship, while 3.2% were uncertain if anyone had done so. No one knew that someone had falsified data while 3.2% was uncertain. No one knew about someone who had plagiarized (data or publications and 1.1% were uncertain). 4.3% knew about someone who had presented results in a misleading manner, while 5.3% were uncertain if anyone at the department had done so.

The PhDs’ attitudes to different forms of scientific dishonesty are presented in Table 2, which also shows results from first-year candidates 2010–20 and PhDs from 2016. 52.8% of the respondents believed that one or more actions that go against generally accepted norms in research integrity were not wrong, that is, the first eight questions in Table 2: Modify, falsify or fabricate data, take credit for the work of others, or repeat analyses until you get statistically significant results. At the same time, respondents were willing to report scientific dishonesty. We compared the attitudes between the finished PhDs and the first-year candidate group through the Kalichman sub-scales and found significant differences with regard to the first two subscales (see Table 2). We report observed mean values, excluding missing observations, so the number of observations for the first-year candidate group is varying a bit, from n  =  494 on subscale 1 to 510 on subscale 4. The analysis showed that the finished PhDs (both 2016 and 2019) scored significantly higher than the first-year candidates on general attitudes and the 2019 PhDs scored significantly higher than the first-year candidates on personal attitudes.

Table 3 shows the PhDs’ assessment of the integrity in their research environment. 5.3% of the PhDs disagreed that their supervisor displayed research integrity (in their own research and in their relations to doctoral students). The PhDs also thought that senior researchers promoted research integrity. 10.6% agreed that research integrity was not promoted in the research group as a whole and 12% reported that they did not know who to ask about research integrity questions.

**Table 3.** Integrity of the Research Environment, PhD 2019. N  =  94.

Response categories Questions	Strongly disagree	Disagree	Neither agree nor disagree	Agree	Strongly agree
1. My main supervisor displayed research integrity in his/her own research	2 (2.1%)	3 (3.2%)	2 (2.15)	23 (24.5%)	64 (68.1%)
2. My main supervisor displayed research integrity in his/her relations with doctoral students	3 (3.2%)	2 (2.1%)	1 (1.1%)	26 (27.7%)	62 (66.0%)
3. Senior researchers in the group where I did my doctoral study promoted research integrity	0	0	9 (9.6%)	31 (33.0%)	54 (57.4%)
4. Junior researchers in the group where I did my doctoral study promoted research integrity	0	1 (1.1%)	14 (14.9%)	26 (27.7%)	53 (56.4%)
5. Research integrity was not promoted in the research group (as a whole) where I did my doctoral studies^1^	42 (44.7%)	30 (31.9%)	12 (12.8%)	8 (8.5%)	2 (2.1%)
6. I knew who to ask if I had a research integrity question	1 (1.1%)	11 (11.7%)	14 (14.9%)	32 (34.0%)	36 (38.3%)

^1^ Reverse scored when forming the Research environment integrity scale.

## Discussion

This follow up study shows that research integrity has been quite stable amongst the PhD candidates at the Faculty of Medicine in Oslo in terms of their attitudes, practices, and knowledge about misconduct. The number of reported instances of misconduct is low and below what has been reported internationally (Fanelli, 2009; Xie et al., 2021). The attitudes are in general in line with good research integrity, but for some issues they are not. This is in particular for trying a variety of different methods of analysis until one is found that yields a result that is statistically significant, to selectively omit contradictory results to expedite publication if confident in the results, and to more truthfully report results in publications than in grant applications.

There are small improvements in attitudes from PhD candidates to finished PhDs for many issues, but not for all. The sub-scale scores for general and personal attitude (Kalichman) show improvements from research fellow to finished PhDs. As there are very few studies on the development in research integrity at the same institution over time, there are few studies to compare with. The general level of breaches with good research integrity (Fanelli, 2009; Xie et al., 2021) indicates that the results do not deviate significantly. It must be noticed though that the results from our previous studies are included in the most recent systematic review and meta-analysis (Xie et al., 2021).

There is little change in the PhDs’ assessment of the research integrity in their environment from 2016 to 2019. This is understandable as integrity takes a long time to change. While it is positive to see that most consider the integrity of the research environment to be good, it is worth noting that more than one tenth agreed that research integrity was not promoted in the research group as a whole and that 12% reported that they did not know who to ask about research integrity questions. This is consistent with the results from the PhD candidates. Many did not know about the existence of relevant policies as PhD candidates and many still are not aware of these when they finish.

The Faculty of Medicine at the University of Oslo has clear policies on research integrity, and training in research integrity is part of the compulsory curriculum at masters and doctoral level. Norway has also had a number of research misconduct scandals that have been covered in the public media.

In general, our results show that attitudes and behaviors in relation to research misconduct have changed very little in the period from 2010–2020, and that there is limited evidence that completing a doctoral programme successfully leads to a positive change in attitudes towards research misconduct. The same goes for participating in research integrity education. This may be because research integrity depends as much on the supervisors, PIs, research group, and research environment as on any formal input (Mumford, Antes, Beeler, & Caughron, 2009; Mumford et al., 2007).

The results are consistent with with previous studies in Norway (Hofmann & Holm, 2016; Hofmann et al., 2013; Holm & Hofmann, 2018), Scandinavia (Hofmann et al., 2020; Hofmann et al., 2015; Jensen et al., 2018; Nilstun et al., 2010), and internationally (Fanelli, 2009; Ljubenković et al., 2021; Xie et al., 2021). Reported breaches of scientific integrity are somewhat higher in our studies than studies in Norway covering multiple disciplines (Elgesem, Jåsund, & Kaiser, 1997; Hjellbrekke et al., 2018; Ljubenković et al., 2021). This is more likely to be because of the low response rate of the transdisciplinary studies than a reflection of less research integrity in medicine and health care.

### Limitations

Despite high response rates, we have to be careful to draw too bold conclusions from the surveys. Those who answer may be the persons with the highest research integrity. However, the relatively high response rate is one of the advantages of this study.

It is likely that researchers are unwilling to disclose their own scientific misconduct, even in anonymous surveys. The practice reported in this study may therefore be underestimated.

Teaching in research integrity has been scored fairly high during these years, so courses may not be the most prominent driver of research integrity.

## Conclusion

For the years 2010–2020 about 1% of the PhD candidates report to have engaged in severe scientific misconduct (FFP) and about 1% report to have presented results in a misleading way. 2.7% report that they know of persons at their department who have engaged in FFP the last 12 months. In total 1.5% report to have experienced pressure to engage in severe scientific misconduct (FFP) while 2.1% report to have experienced pressure to present results in a misleading way. On average 12.8% report to have been exposed to unethical pressure concerning inclusion or ordering of authors during the last 12 months, and 28.8% report to have knowledge about their department's written policies about research integrity. While some attitudes improve over the years, attitudes in general are not much changed from 2010–2020. While none of the PhDs that received a PhD from the Faculty of Medicine at the University of Oslo in 2019 reported to have engaged in FFT or having experienced pressure to do so, 26.6% of respondents had experienced unethical pressure in relation to authorship during the course of the PhD fellowship. 4.3% knew about someone at their department who had presented results in a misleading manner, and some attitudes were not in line with traditional conceptions of research integrity, but most agreed that their research environment displayed research integrity.

This long-term follow up study shows that few PhD-candidates report to engage in severe scientific misconduct, that they experience little pressure to do so, and with some exceptions, attitudes in in line with good research integrity. However, pressure in relation to authorship is relatively common. There is some improvement in research integrity from PhD candidates to recently finished PhDs, but in general, research integrity is stable over time. Improving research integrity is a difficult, but important task.

### Educational Implications

Science ethics education and research integrity training are important, but not sufficient for improving research integrity. Strong role models and local norms may undermine the effect of good research integrity programs. Therefore, educational efforts should not only be directed towards PhD candidates, but also towards supervisors, senior scientists, and research role models.

### Best Practices

Efforts to improve research integrity promoting programs and education should be continued, but combined with adapted measures to target supervisors, research leaders, and scientific role models.

### Research Agenda

The study indicates that we need more knowledge about the influence of supervisors, senior researchers, and role models compared to the influence of science ethics education and research integrity training.
